# Artificial Intelligence and Clinician Burnout in the United States: A Narrative Review

**DOI:** 10.7759/cureus.112814

**Published:** 2026-07-16

**Authors:** Crissinee Sucharitrak

**Affiliations:** 1 Business Analytics, California Baptist University, Riverside, USA; 2 Nursing, Santiago Canyon College, Orange, USA

**Keywords:** ambient artificial intelligence scribe, artificial intelligence, clinical decision support, clinician burnout, documentation burden, electronic health record, healthcare workforce, large language models, nursing burnout, physician burnout

## Abstract

Burnout remains one of the defining occupational hazards of healthcare in the United States, and it spares no one on the care team: physicians, nurses, trainees, and assistants all report it at high rates. When generative AI entered healthcare around 2023, it arrived quickly and carried a promise of relief from the administrative burden that so many clinicians identify as a reason for their exhaustion. This review asks one question about that promise: Does the current evidence from the United States indicate that AI alleviates or worsens clinician burnout, and under what implementation conditions? We approached this question through a structured thematic narrative synthesis, weighting evidence by study design. Randomized controlled trials carried the greatest interpretive weight, while non-randomized studies were considered lower-certainty supporting evidence. The first randomized trials of AI-assisted scribing, published in 2025-2026, are encouraging with respect to documentation time and, in some trials, well-being. Yet most of what has been published so far is non-randomized, single-center, and short-term. The more troubling pattern is that AI tends to displace work rather than reduce it. Tasks are not eliminated but moved: from production to oversight, and potentially from physicians to nurses and assistants. Where earlier commentaries offered the metaphors of a "double-edged scalpel" and a "productivity paradox," this review proposes "displacement of burden" as a framework that can actually be tested.

## Introduction and background

What burnout is, and why it persists

The phenomenon of burnout has been described in terms of three factors: emotional exhaustion, depersonalization or cynicism, and reduced personal accomplishment. It is now very common among healthcare professionals in the United States, including doctors, nurses, residents, and assistants [1-9]. As it is associated with medical errors, workforce turnover, and professional distress, it is now a patient safety and workforce sustainability issue, not merely a wellness issue [10-15]. The magnitude of the problem, its causes, and effects are discussed in the sections "The Persistence of Burnout in United States Healthcare" and "Why Burnout Matters: The Case for Intervention".

Why this review, and why now

One of the most consistent and well-established contributors to clinician burnout is the electronic health record, together with its associated documentation and clerical burden, a driver that, along with its reframing as "moral injury," we examine in the section "The Documentation Era: How the Electronic Health Record Set the Stage for AI". This is precisely the problem that AI vendors and healthcare organizations seek to address. By 2023, ambient AI dictation, automatic drafting of patient messages, automated coding, and AI-assisted triage had moved beyond piloting into full enterprise implementation [16]. The speed of implementation has outpaced the evidence base, which currently diverges into good news and bad news. Two recent commentaries describe the effects of AI on burnout as a "double-edged scalpel" and a "productivity paradox" [17,18]; yet neither discourse weighs the strength of the evidence for each position, nor what becomes of the displaced burden. This review therefore asks a single question: What does the current United States evidence indicate about whether AI alleviates or worsens clinician burnout, and under what implementation conditions? Three subsidiary objectives follow from this question: to synthesize the first randomized trials of ambient AI, to situate that evidence within the quality-improvement and observational literature, and to develop a burden-displacement framework that explains when relief is real and when it is merely relocated.

## Review

Materials and methods

The literature search was carried out in the PubMed/MEDLINE database, with a supplementary manual search of the reference lists of included studies. The last literature search was performed in June 2026. Articles published from 2016 to 2026 were included; there were no restrictions on publication type beyond the requirement for English-language text, with particular emphasis on the generative AI era (2023-2026). Search terms included free-text terms associated with clinician, physician, and nurse burnout, together with terms related to electronic health records, AI and generative AI, ambient documentation and ambient AI scribes, clinical decision support systems, large language models, AI-drafted patient messages, automated medical coding, and AI-assisted triage.

Eligible sources were those concerning clinician burnout and the use of AI in clinical settings. Eligible source types included research studies, systematic reviews, quality-improvement or observational studies, and commentaries, with preference given to sources published in the United States and to open-access papers. Non-English sources, non-peer-reviewed material, sources irrelevant to clinician burnout or AI in clinical practice, and duplicate sources were not considered. A two-stage screening process was used: first, title and abstract screening, followed by full-text review against the inclusion and exclusion criteria. In total, 73 sources were included in this review. By study design, these sources comprised randomized controlled trials, quality-improvement or observational studies, systematic reviews and meta-analyses, and commentaries or frameworks. 

Data extracted from the sources included the type of AI tool, the clinical setting, the study design, measures of burnout, well-being, and documentation burden, and the implementation context. A structured thematic narrative synthesis was conducted, guided by the Scale for the Assessment of Narrative Review Articles (SANRA). No formal risk-of-bias tool was applied. Instead, evidence was weighted by study design, and each source type had a predefined role. Randomized controlled trials served as the primary basis for every outcome-related conclusion. Quality-improvement and observational studies were treated as convergent but lower-certainty supporting evidence, and they are labeled as such wherever they appear in the review. Systematic reviews and meta-analyses were used to check the direction and consistency of effects. Commentaries and conceptual frameworks informed the background and framing only; none was used as outcome evidence. No meta-analysis was conducted, and a qualitative synthesis of the evidence was carried out. All references were selected independently and cross-checked against their original sources via Crossref.

Review

The Persistence of Burnout in United States Healthcare

Any argument about how AI can “solve” burnout should consider the scale of the issue. The proportion of United States physicians experiencing burnout has changed over time due to various factors outside the healthcare system, but it has remained consistently high compared with that of other professions in the United States [1,2]. Among nurses, the prevalence of burnout and its role in contributing to turnover have been clearly demonstrated in large cross-sectional United States studies [3], consistent with an already established trend of dissatisfaction and intention to leave, especially among direct-care nurses [4]. Trainees are likewise affected, as clinical specialty and working environment have been linked to burnout and career-choice regret among United States resident physicians [5]. Workload predicts burnout and intent to leave across the entire healthcare workforce, physicians, nurses, and non-physician clinical and non-clinical staff, and not only among higher earners [12].

The assistive and allied workforce warrants emphasis here because this is the sector where the redistributive effect of AI (discussed in Section "Implementation: What Determines Which Edge Wins") is most likely to manifest. Burnout and attrition rates among acute-care CNAs were very high during the pandemic due to understaffing, lower pay, and lack of recognition [6]; Long-term care nursing assistants reported high burnout and low compassion satisfaction, but no significant difference in intent to leave [7]; in a national sample survey, 32.4% of U.S. emergency nurses had left their job or were no longer working, with burnout cited by 10.6% as a reason for leaving, ranking below better pay elsewhere and insufficient staffing [8]; and a majority of pharmacists in health systems reported high burnout rates during the pandemic [9]. It is this demographic whose voice is conspicuously missing from research on AI and burnout (Table 1). It is far from accidental that the support and allied services workforce happens to be both the most overstretched and the least researched in relation to the current paper’s argument, since it is precisely in this area that displacement takes place most frequently (discussed in Section "Synthesis: The Displacement of Burden*"*).

**Table 1:** Burnout and turnover across the United States care team. Bracketed numbers refer to the reference list; values are reported as presented in each cited source.

Provider group	Burnout/turnover metric	Reported finding	Ref
Physicians	At least one burnout symptom	45.2% (2023); peak of 62.8% (2021)	[1]
Registered nurses	Burnout among those leaving/considering leaving	31.5% / 43.4%	[3]
Resident physicians	Burnout; career-choice regret	~45%; ~14%	[5]
U.S. emergency nurses	Job turnover (left job or not currently working); burnout as a cited reason	32.4% (vs 28.7% of other nurses, not significant); burnout 10.6%	[8]
Health-system pharmacists	Reported increased burnout during the COVID-19 era	78.20%	[9]
Long-term-care nursing assistants	Higher burnout and lower compassion satisfaction	Elevated; no significant difference in intent to leave	[7]
Certified nursing assistants (acute care)	High burnout and turnover associated with staffing, pay, and recognition	Qualitative/elevated	[6]

Why Burnout Matters: The Case for Intervention

The link between burnout, safety, and workforce loss is what elevates burnout above a morale problem. In a survey of more than 6,500 United States physicians, burnout was associated with more than double the risk of a recent significant medical error, independent of work-unit safety grade [11], and resident burnout has been linked to poor working conditions and a higher risk of errors [10]. Apart from safety issues, longitudinal research has shown that burnout leads to decreased levels of professional effort among individuals [13], aggravating shortages already predicted among nurses and assistants [8,12]. The annual cost of physician burnout in the United States is estimated to be approximately $4.6 billion, mainly because of turnover and absenteeism [14]. The personal cost, in turn, is associated with increased risks of depression, anxiety, and even suicide [15]. Framed this way, the case for intervention rests not on morale but on the two outcomes a health system can least afford to lose, patient safety and a stable workforce, against which any tool marketed as a remedy for burnout must ultimately be judged.

The Documentation Era: How the Electronic Health Record Set the Stage for AI

AI was not introduced into an unblemished field but into one beset by documentation burden, a problem that the electronic health record has helped to create. A traditional time-and-motion study showed that for every hour spent on direct patient care, doctors spend two hours on the electronic health record and associated paperwork, along with additional documentation time at home. It is this standard that makes documentation relief a highly coveted solution [19]. Meta-analytical evidence indicates that electronic health record use is associated with professional burnout among healthcare practitioners, especially after-hours electronic health record use, which increases the risk even further [20], while high-level decision support adds to the cognitive burden through alert fatigue [21]. Of particular importance for AI policy considerations is the idea that such stress may be considered moral injury [22]; when the problem involves elements of values and control, a technology that increases productivity but decreases the sense of control may actually lead to reduced well-being. This distinction matters for what follows: if documentation burden is the proximate target of most AI tools, an honest appraisal must ask not only whether documentation time is shortened but also whether the sense of control implicated in moral injury is restored rather than further eroded [22].

Risks: How AI Can Worsen Clinician Burnout (Edge One)

The first edge of the blade is that AI can introduce new burdens even as it removes old ones.

Automation bias and complacency: When healthcare practitioners rely on AI algorithms, they become vulnerable not only to their own errors but also to a subtler, more insidious erosion of vigilance. The risks of automation bias and the potential for patient harm from AI decision-support algorithms have been detailed in the literature [23], as has the impairment of diagnostic reasoning caused by erroneous suggestions from language models in a randomized trial involving clinicians well versed in AI literacy [24].

The oversight burden and invisible labor: Despite the efficiency of drafting tools, such software still requires validation, which can itself be labor-intensive. Literature on AI and physician autonomy points out that although some work is routinized, auditing work can increase [25]. The invisible work necessary for the safe implementation of AI has been described in the qualitative literature [26].

Deskilling and professional identity: The use of AI over an extended period may increase the risk of skill degradation among healthcare professionals, as found in scoping and mixed-method reviews; for example, decreased capability to detect adenomas in patients undergoing colonoscopy after discontinuation of AI assistance [27,28]. Moreover, the use of AI could be viewed by healthcare professionals as a threat to their professional identity and result in resistance, including fears of being replaced in the job market, particularly among radiologists, with such concerns reported in up to 38% of cases [29,30,31].

Real-world failure: The deployment of poorly performing technologies creates additional burden and lowers trust. For instance, an external validation of a clinically used sepsis prediction algorithm reported an area under the curve of 0.63, compared with the range of 0.76-0.83 reported by the system's developers [32]. Such discrepancies can create false alerts that need to be addressed, representing yet another example highlighted in Section "Synthesis: The Displacement of Burden*"*. Across these four pathways, automation bias, oversight labor, deskilling, and system failure, the common underlying problem is the potential for work removed by AI to reappear in another form, as described in Section "Synthesis: The Displacement of Burden*"*.

Benefits: How AI Can Relieve Clinician Burnout (Edge Two)

The second edge is that, when deployed well, AI targets precisely the documentation and messaging burden most strongly implicated in burnout.

Ambient AI scribes - the emerging randomized evidence: The most important recent development is that ambient documentation has begun to be tested in randomized trials rather than in enthusiastic pilots alone. A randomized academic-center trial showed a decrease in note time of about 9.5%, some reductions in burnout and workload, and the generation of clinically significant errors that required revision by physicians [33]. Another pragmatic stepped-wedge randomized trial showed decreases in practitioner exhaustion and disengagement [34], while a randomized crossover trial involving two scribe tools showed decreased burnout and note time [35]. Their recency relative to the commentary literature makes these trials the crux of this review.

Quality-improvement and observational signal: Beyond the randomized trials, uncontrolled quality-improvement and observational reports from United States health systems point in the same direction. These designs lack randomization and concurrent controls, so the figures that follow should be read as convergent, lower-certainty signals rather than established effects. For example, a multi-system quality-improvement initiative reported a decrease in burnout from 51.9% to 38.8%, along with a decreased task load and approximately 11 min saved per day on after-hours documentation [36]. Single-system studies include reports of decreased mental workload and improved well-being [37,38], decreased cognitive load [39], and time savings [40]. Pediatric physicians experienced a decrease in burnout from 54.9% to 33.3% [41], whereas family medicine residents achieved savings of about 3 min per patient on documentation and improved well-being [42]. Cohort studies reported decreases in electronic health record use and note times, but decreases in after-hours note times occurred only in subcohorts [43]. Implementation studies examined processes and user perspectives [44,45], while systematic reviews and a meta-analysis included studies of documentation burden and found a moderate reduction in burden (standardized mean difference = -0.71), with high heterogeneity [46-48].

Beyond the note - inbox, triage, coding, and decision support: Quality-improvement and systematic review research has examined the use of generative AI to draft responses to messages within patient portals, an important contributor to clinician burnout. A single-group quality-improvement study showed that using AI to generate responses to messages had no effect on time but significantly decreased workload and exhaustion [49]. Improvements in the perceived efficiency and empathy of communication were reported, although the use of generative drafts was limited [50]. Systematic reviews found that AI-generated drafts were comparable to human responses in terms of empathy while also identifying potential issues, including incorrect drafts [51-54]. Other applications included message triage [55,56], prior authorization review, where denials might be automated [57], medical coding [58,59], decision support systems aimed at preventing false positives [60], and nursing practice [61]. Taken together, these results suggest that “artificial intelligence for burnout” represents a family of interventions rather than a single solution, with each differing in its evidence base and associated risks (Table 2). What distinguishes this body of literature from what has gone before is its convergence: although there may be some variation in effect sizes, randomized trials, quality-improvement studies, and observational data from various disciplines and institutions point toward the same overall direction [46-48].

**Table 2:** AI applications and their relationship to clinician burnout. Bracketed numbers refer to the reference list.

AI application	Mechanism	Relationship to burnout	Strongest evidence type
Ambient AI scribes	Automatically draft clinical notes from encounters	↓ documentation time and burnout; residual error oversight required	Randomized controlled trials [33-35]; quality-improvement and observational studies [36-43]
AI-drafted patient messages	Draft replies to portal messages	↓ workload and exhaustion; oversight required for unsafe drafts	Single-group quality-improvement study [49]; reviews [51-54]
Message triage	Prioritize and route inbox messages	Potential reduction in workload; accuracy oversight required	Experimental and comparative studies [55,56]
Prior authorization	Automated review of denials	Risk of displaced burden and appeals	Analysis [57]
Automated medical coding	Large language model-assisted code assignment	Improved efficiency; validation required	Model studies [58,59]
Clinical decision support	Risk prediction and alerts	Mixed: alert fatigue versus fewer false alarms	Clinical decision support studies [21,60]
Nursing applications	Operational and clinical AI in nursing	Emerging; understudied in relation to burnout	Integrative review [61]

Implementation: What Determines Which Edge Wins

Whether AI mitigates or worsens burnout may be less a matter of the technology's potential and more a matter of how it is deployed, assessed, and regulated. The same virtual assistant may lighten the load in one clinic but increase the burden in another. Two broad themes contextualize this debate: over-reliance on technology may encourage automation complacency [62], and poorly designed systems can themselves contribute to burnout [63].

Organizational readiness and workflow fit: Implementation science research stresses the importance of organizational readiness, workflow compatibility, training, and support during implementation rather than the purely technical capabilities of the tool [64,65,66]. Innovation assessments and qualitative studies of ambient documentation technology in ambulatory care settings show that adoption and value generation are highly dependent on compatibility with existing workflows and the potential for scalable usability [44,45,48].

Trust, oversight, and the human in the loop: Trust in AI-based clinical decision support technologies among healthcare providers is inconsistent and depends on the transparency and reliability of the technology [67]. Excessive trust in the technology can lead to automation complacency [62], while insufficient trust may impose an additional oversight burden. Therefore, approaches to governance are moving from a simple "human in the loop" paradigm toward participatory governance [68], frameworks for marketed AI/machine learning medical devices [69], and specific recommendations for responsible clinical decision support [70].

Measuring what matters - burnout-aware evaluation: Most implementations are evaluated based on efficiency rather than clinician well-being; systematic reviews call for the use of burnout and well-being metrics alongside multisite, long-term evaluations [63,46-48]. Where AI has been introduced to address a specific constraint, such as critical care understaffing, qualitative research has shown improved working conditions [71]. The crucial factor here is whether the implementation strategy was developed with workers' well-being in mind. The practical corollary is that the same tool may be reported as a success or a failure depending on what is measured and who is asked; until well-being endpoints and the experiences of the entire care team are routinely captured, the literature will continue to under-detect the costs that fall outside the physician's note [63].

Synthesis: The Displacement of Burden

**Figure 1:**
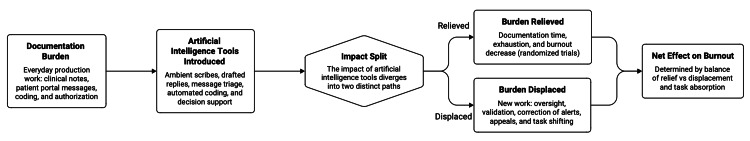
The displacement-of-burden framework. AI converts production work, such as documentation, into oversight and validation work. The net effect on clinician burnout depends on whether the burden relieved exceeds the burden displaced and on who absorbs the remaining burden. Image created by the author.

On the risk-benefit continuum, the common theme that emerges is that AI replaces work more often than it eliminates it: documentation effort is converted into oversight and validation work [25,26]. It remains to be seen whether the oversight burden is less than the displaced burden and therefore improves well-being. Reframing the issue in terms of "burden displacement" extends the ideas behind the metaphors of the "double-edged scalpel" and the "productivity paradox" [17,18] by defining both the mechanism and the conditions of burden relief. Figure 1 summarizes this framework.

Actual case studies of failure demonstrate that the mechanism works as expected. A poorly calibrated sepsis model produces false positives whose correction requires manual effort [32], and automated prior-authorization review may shift effort toward appeals [57]. In either scenario, the burden is neither relieved nor reduced; it is merely displaced.

Burden displacement also has a directional and an equity dimension: when physicians' documentation burden is reduced, the remaining work might be passed to nurses and assistant staff, who are among the most burned-out yet least studied groups [6-9]. Biased algorithms, in turn, might shift harm toward marginalized patients [72,73]. The lack of attention to the latter in the AI-and-burnout literature is not just an omission; it reflects a lack of attention to the question of who bears the burden. Read as a whole, then, the evidence supports neither the optimistic nor the alarmist account in its strongest form, but rather a conditional one, in which the direction of AI's effect on burnout is determined less by the algorithm than by the surrounding choices regarding workflow, governance, measurement, and equity.

The conclusions are therefore qualified: the success of AI depends on whether the burden it alleviates outweighs the burden of supervision and governance it introduces and whether its impact on the well-being of all members of the care team is measured.

Future Directions

The synthesis highlights a number of areas on which future research should focus. To start with, there is a need for large-scale, multicenter randomized controlled trials with sufficient follow-up time to identify clinician well-being as a critical endpoint, rather than focusing only on efficiency [33-35,46-48,63]. Next, the evaluation process must include not just physicians but the entire care team. Multicenter randomized or stepped-wedge trials with prespecified, team-level endpoints, including nurse and assistant workload and well-being, would directly test whether displaced tasks fall on personnel who remain relatively understudied [6-9]. Time-motion studies could then trace where oversight and validation tasks actually settle after deployment. In addition, prospective studies examining governance and trust measures are needed to ensure that oversight is proportionate and does not simply conform to the "human-in-the-loop" paradigm [67-70]. Moreover, future deployments should be studied for their effects on equity among patients and staff to determine whether algorithmic bias and task displacement disproportionately affect marginalized patients and lower-wage staff [72,73]. For reference, the study design and primary focus of all cited sources are summarized in Table 3.

**Table 3:** Characterization of all cited sources. Bracketed numbers refer to the reference list.

Source (author, year)	Study / evidence type	Focus / contribution
Shanafelt TD et al. (2025) [1]	Longitudinal survey	Physician burnout prevalence, 2011 to 2023
Shanafelt TD et al. (2022) [2]	Longitudinal survey	Physician burnout during coronavirus disease 2019
Shah MK et al. (2021) [3]	Cross-sectional survey	Nurse burnout prevalence in the United States
McHugh MD et al. (2011) [4]	Cross-sectional study	Nurse dissatisfaction/burnout and patient care
Dyrbye LN et al. (2018) [5]	Survey	Specialty, burnout, and career-choice regret (residents)
Snyder RL et al. (2023) [6]	Qualitative/observational	Certified nursing assistant burnout and turnover (coronavirus disease 2019)
Richert M, Zucchero R (2023) [7]	Survey	Long-term-care nursing assistant burnout
Norful AA et al. (2023) [8]	National survey	Emergency-nurse burnout and turnover (United States)
McQuade BM et al. (2022) [9]	Survey	Health-system pharmacist burnout
Surawattanasakul V et al. (2024) [10]	Cross-sectional study	Resident burnout, conditions, and medical errors
Tawfik DS et al. (2018) [11]	Survey	Physician burnout and self-reported errors
Rotenstein LS et al. (2023) [12]	Survey	Work overload, burnout, and intent to leave
Shanafelt TD et al. (2016) [13]	Longitudinal study	Burnout and reduced professional work effort
Han S et al. (2019) [14]	Economic analysis	Attributable cost of physician burnout
Ryan E et al. (2023) [15]	Systematic review	Burnout, depression, anxiety, and suicidality
Poon EG et al. (2025) [16]	Survey	Adoption of AI in healthcare
Bouguettaya A, Aboujaoude E (2026) [17]	Commentary	AI and burnout: the double-edged scalpel
Goodson DA et al. (2025) [18]	Commentary	AI and burnout: the productivity paradox
Sinsky C et al. (2016) [19]	Time-and-motion study	Physician time: about two hours of electronic health record work per hour of patient care
Wu Y et al. (2024) [20]	Systematic review/meta-analysis	Electronic health record use and burnout; after-hours use raises risk
Jankovic I, Chen JH (2020) [21]	Review	Clinical decision support and burnout (alert fatigue)
Williams MS (2021) [22]	Commentary	Electronic health record distress as moral injury
Khera R et al. (2023) [23]	Commentary	Automation bias and harm from AI-driven decision support
Qazi IA et al. (2026) [24]	Randomized controlled trial	Flawed large language model advice impairs clinician diagnostic reasoning
Grosser J et al. (2025) [25]	Scoping review	AI and physician autonomy; oversight burden
Ulloa M et al. (2022) [26]	Qualitative study	Invisible clinical labor required to integrate AI
Heudel PE et al. (2026) [27]	Scoping review	AI deskilling and loss of expertise
Natali C et al. (2025) [28]	Mixed-method review	AI-induced deskilling in medicine
Jussupow E et al. (2022) [29]	Survey	Professional-identity threat and resistance to AI
Ackerhans S et al. (2025) [30]	Experimental study	Identity threat and trust in AI decision support
Huisman M et al. (2021) [31]	International survey	Radiologists' fear of replacement (about 38%)
Wong A et al. (2021) [32]	External validation	Proprietary sepsis model: area under the curve 0.63 in practice (false alarms)
Lukac PJ et al. (2025) [33]	Randomized controlled trial	Ambient scribe: note time down about 9.5%, modest burnout reduction, residual errors
Afshar M et al. (2025) [34]	Pragmatic randomized controlled trial	Ambient AI: reduced exhaustion and disengagement
Chowdhury A et al. (2026) [35]	Randomized crossover trial	Two ambient scribes: reduced burnout and note time
Olson KD et al. (2025) [36]	Quality improvement (multi-system)	Burnout 51.9% to 38.8%; about 11 minutes per day saved
Stults CD et al. (2025) [37]	Evaluation	Ambient AI documentation platform
Shah SJ et al. (2025) [38]	Observational study	Ambient scribe: usability, documentation burden, burnout
Hudson TJ et al. (2025) [39]	Observational study	Ambient AI and cognitive load
Guo Y et al. (2026) [40]	Evaluation	Ambient AI: efficiency and documentation burden
Pelletier JH et al. (2025) [41]	Pilot study	Pediatric: burnout 54.9% to 33.3%
Anderson W, Koran-Scholl JB (2026) [42]	Pilot study	Resident ambient AI: documentation time and well-being
Pearlman K et al. (2025) [43]	Cohort study	AI scribe and electronic health record efficiency (after-hours subgroup)
Lawrence K et al. (2026) [44]	Innovation evaluation	Implementing ambient documentation for ambulatory clinicians
Van Tiem J et al. (2026) [45]	Qualitative study	Clinician perspectives on ambient AI scribes
Zhao J et al. (2025) [46]	Systematic review/meta-analysis	AI tools and documentation burden (standardized mean difference -0.71)
Bracken A et al. (2025) [47]	Systematic review	AI-powered documentation systems
Ohde JW et al. (2026) [48]	Review	Barriers and opportunities of scaling ambient AI scribes
Garcia P et al. (2024) [49]	Single-group quality-improvement study	AI-drafted patient replies: reduced workload and exhaustion
English E et al. (2024) [50]	Survey	Utility of AI-generated draft replies
Baxter SL et al. (2024) [51]	Review	Generative AI replies to patient messages
Hu D et al. (2025) [52]	Systematic review	Generative AI for drafting patient-message responses
Mandal S et al. (2025) [53]	Observational study	Utilization of generative AI-drafted responses
Biro JM et al. (2025) [54]	Analysis	Opportunities and risks of AI in patient-portal messaging
Pham JH et al. (2024) [55]	Experimental study	Large language model triage of inbox messages
Alsumait A et al. (2025) [56]	Comparative study	AI versus human triage of clinic messages
Raza S et al. (2026) [57]	Analysis	AI in prior-authorization review
Hou Z et al. (2025) [58]	Model study	Fine-tuned large language models for medical coding
Naliyatthaliyazchayil P et al. (2025) [59]	Model study	Large language model reasoning for coding and readmission risk
Gupta A et al. (2024) [60]	Decision-support study	Sepsis clinical decision support minimizing false alarms
Hassanein S et al. (2025) [61]	Integrative review	AI in nursing: clinical and operational impacts
Saadeh MI et al. (2025) [62]	Commentary	Automation complacency
Sarraf B, Ghasempour A (2025) [63]	Systematic review	AI and electronic health record-related burnout
Jacob C et al. (2025) [64]	Systematic review	Implementation framework for long-term real-world impact of AI tools
van de Sande D et al. (2024) [65]	Framework	AI models require multifaceted implementation evaluation
Nair M et al. (2025) [66]	Framework	Critical activities for AI implementation and adoption
Tun HM et al. (2025) [67]	Systematic review	Trust in AI-based clinical decision support among healthcare workers
Griffen Z, Owens K (2024) [68]	Commentary	Participatory governance of AI (human in the loop)
Babic B et al. (2025) [69]	Framework	Governance of marketed AI/machine-learning medical devices
Labkoff S et al. (2024) [70]	Recommendations	Responsible AI-enabled clinical decision support
Bienefeld N et al. (2025) [71]	Qualitative study	AI to alleviate shortages in critical care
Cary MP Jr et al. (2023) [72]	Scoping review	Mitigating bias in clinical algorithms (health equity)
Nazer LH et al. (2023) [73]	Review	Bias in AI algorithms and mitigation

Limitations

There are certain limitations associated with this analysis. First, the review is not systematic; the lack of a prospectively defined search strategy, a formal risk-of-bias assessment, and the application of Preferred Reporting Items for Systematic Reviews and Meta-Analyses (PRISMA) criteria for study selection contributes to the risk of both selection bias and citation bias. Second, only one author carried out the review; however, all citations were checked against the original sources. Third, the review covers only the United States healthcare system and, thus, may not be generalizable to other systems. Fourth, there is a lack of randomized evidence; the vast majority of the included studies are observational or cross-sectional and use various instruments and measures of burnout, making any meta-analysis impossible. Finally, because AI is a rapidly developing field, some of the conclusions may become less relevant as the technology advances. In addition, the search was conducted primarily in PubMed/MEDLINE and supplemented by manual searching of reference lists; therefore, relevant studies indexed only in other databases, such as Embase, CINAHL, Scopus, or Web of Science, may have been missed. The inclusion of heterogeneous study designs also carries a risk of bias, and findings from non-randomized studies may overestimate the benefits of AI.

## Conclusions

AI is neither a complete solution to the problem of clinician burnout nor simply an additional task; rather, it represents a shift in labor, the outcomes of which depend on how it is implemented. Randomized trials of ambient documentation are genuinely promising with respect to documentation time and, in some trials, well-being. The larger body of supporting evidence, however, remains observational, single-center, and short-term, and its more dramatic findings should not be interpreted as established effects. The greatest common threat is not failure itself but the shifting of labor from production to oversight, as well as the redistribution of burden from physicians to nurses and assistants, whose experiences are less frequently quantified. The development of this field requires comprehensive, multicenter, longitudinal research that captures well-being indicators for all members of the care team. It also requires governance that keeps oversight proportionate and, most importantly, sustained attention to equity.
